# Effectiveness of a Ward level target accountability strategy for hand hygiene

**DOI:** 10.1186/s13756-019-0641-0

**Published:** 2019-11-15

**Authors:** Surinder M. S. Kaur Pada, Poh Ling Chee, Sarathemani Rathenam, Kim Sim Ng, Lilibeth Silagan Alenton, Lishi Poh, Paul Anatharajah Tambyah

**Affiliations:** 1 Ng Teng Fong General Hospital and Jurong Community Hospital, Jurong Health Campus, National University Health Services, 1 Jurong East Street 21, Singapore, 609606 Singapore; 2 National University Hospital, National University Health Services, 5 Lower Kent Ridge Rd, Singapore, 119074 Singapore

**Keywords:** Hand hygiene, Accountability, Target, Ward

## Abstract

**Background:**

Hand hygiene is a simple and effective solution in prevention of Multi Drug Resistant Organisms. Hand Hygiene campaigns have mostly taken the form of a generalised hospital approach with visual reminders and rewards for improvement in compliance. We describe a hand hygiene programme that sets an individualised ward target to increase accountability and drive improvement.

**Methods:**

We undertook to develop a “Hand Hygiene Accountability” model, where the mean compliance rate, using the WHO hand hygiene assessment tool, for each ward over the past 6 months plus 10% was used as a target for that particular ward.

Rewards were given to wards with the most percentage improvement over the year. A graded escalation was used for wards that did not meet targets based on 1,2 or 3 months of non-compliance. The most extreme action, setting up a task force directed by the Chairman of our Medical Board, would be required if 3 continuous months of non-compliance was observed. Hand Hygiene audits were performed by staff trained using the WHO audit tools. The same strategy was repeated at our community hospital.

Active surveillance testing for Methicillin Resistant *Staphylococcus aureus* (MRSA) using nasal, groin and axilla swabs established before the project continued to be in operation, as did surveillance for hospital acquired MRSA bacteraemia (using NHSN criteria), hospital-onset Clostridioides difficile (HO-CD), and multi-resistant gram-negative bacilli.

**Results:**

Data from July 2015 to December 2017 was analysed. In the acute and community hospitals, 21,582 and 5770 hand hygiene (HH) observations were undertaken respectively.

In the acute care hospital, HH compliance rates went from 65 to 78% (*p*-value < 0.00001). There was a reduction in MRSA bacteraemia from 5 episodes at the start of the study to 0 in 2017.

In the community hospital, HH compliance improved from a mean of 64 to 75% (*p*-value 0.00005). MRSA transmission rate decreased from 5.72 per 1000 patient days, to 2.79 per 1000 patient days (*p*-value 0.00035) with an admission prevalence of 13.1% for 2016 and 20.6% in 2017.

**Conclusions:**

Using a ward level accountability for hand hygiene is possible and can be successful in improving hand hygiene rates, possibly reducing transmission of MDROs. Realistic targets need to be set and adequate rewards and incentives provided to ensure continuous improvement.

## Background

Each year, large numbers of patients around the world are affected by health care-associated infections (HCAIs). The WHO Hand Hygiene guidelines and field tested toolkits are well established worldwide, but implementation of these guidelines remains challenging especially in resource limited settings [1–4].

The evidence that hand hygiene is a simple and effective solution in prevention of multi-drug resistant organism (MDRO) transmission is strong. Improvements in hand hygiene compliance and/or increased alcohol based hand rub (ABHR) consumption have been associated with substantial decreases in MDROs’ infection and/or colonization rates, mainly for MRSA [5].

Hand Hygiene campaigns, with visual reminders and various forms of rewards for improvement in compliance have been widely used globally [6–9].

Unfortunately, campaign fatigue may lead to diminished hand hygiene rates [10]. The need to maintain innovative strategies to engage Health Care workers (HCWs) is a constant challenge to most Infection Control Teams.

We describe an intervention that we undertook in our 700 bedded acute care hospital (ACH), which we replicated in our 400 bedded community care hospital (CCH) located on the same site, that resulted in a sustained improvement in Hand hygiene rates for more than 1 year after initiation.

## Methods

We had recently moved, from a 350 bedded hospital, into a new facility of 700 bed ACH and a 400 bed CCH in June 2015. As such, many freshly graduated nurses and allied health personnel from a number of different countries and educational backgrounds were deployed across various wards. Hospital specific infection control training was largely confined to a brief orientation session together with a number of other topics.

A hospital wide target for Hand Hygiene, was to be used as a Key Performance Indicator by the hospital administration. However, we felt that setting a hospital wide Hand Hygiene rate would not help us accomplish our task in improving Hand Hygiene and achieving better outcomes for our patients [11].

Instead, we undertook to develop a “Hand Hygiene Accountability” model, where the mean compliance rate using the WHO hand hygiene assessment tool for each ward over the past 6 months was used as a baseline. We then added 10% to this mean and used that as a target for that particular ward. This meant that each ward was accountable for their own hand hygiene rate and realistic targets based on a 10% improvement was set.

Additional components included rewards for the wards that had achieved the most percentage improvement (rather than the highest Hand Hygiene rate) over the year. For wards that did not meet targets, we used a graded response. If targets were not met in the first month, our Infection Control Nurses (ICN) would speak to the unit managers to step up unit level education. Two months of failure to reach targets resulted in efforts from the infection control committee chair and more intense education. In the case of 3 consecutive months of performance below target, a ward level task force supported by the Chairman of the Medical Board with the help of ICN, was established to devise a comprehensive strategy for improvement. Hand Hygiene champions, one doctor and one nurse, per ward were pre-emptively identified on every ward who would be relied upon to lead a task force if needed.

**Fig. 1:**
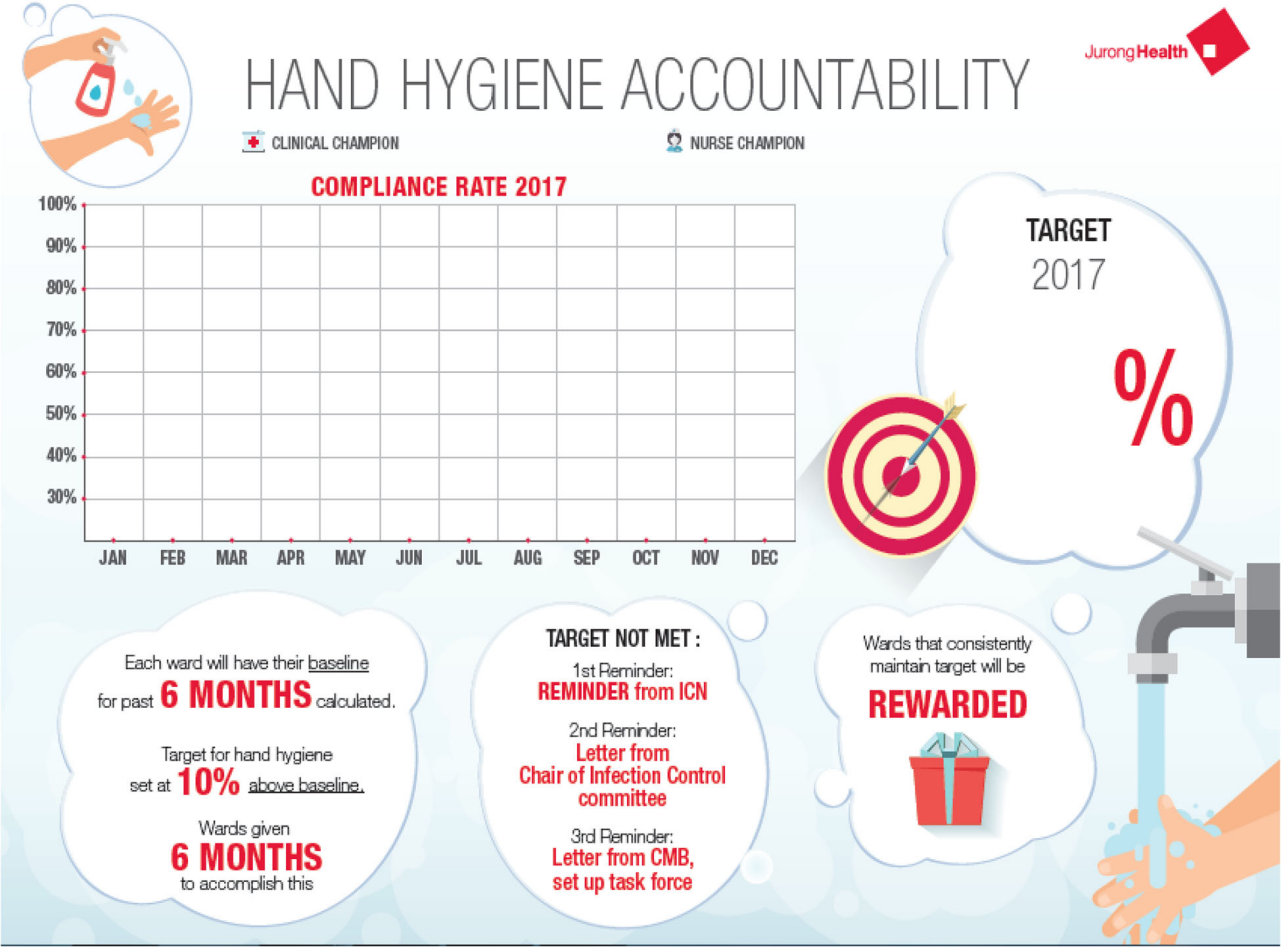
Poster used to summarise individualised targets, put up in every ward

Posters were put up in staff areas on every ward showing their specific target compliance rates over the year, with color-coded indicators - green indicating achievement of targets, and red for failure to achieve the desired target (Fig. 1).

Hand Hygiene audits were performed by staff trained using the WHO audit tools including “Secret Shoppers”(who were administrative staff not known to ward staff) in addition to those done by our infection control liaison nurses (ICLNs) and ICNs. Apart from a Hand Hygiene Day event, no other projects or interventions were performed at this time.

We performed MRSA screening by taking one swab from the nostrils, which was rotated gently in both nostrils, and one swab from the axilla and groin. We used flocked nylon fibre tip swabs with 1 mL of Amies transport medium (Copan Italia SpA, Brescia, Italy). The sample was plated by the BD Kiestra™ InoqulA™ (BD Kiestra B.V., Drachten, The Netherlands) directly on OXOID Brilliance™ MRSA 2 agar (Thermo Fisher Scientific, Perth, UK) or BBL™ CHROMagar® MRSA II (Becton, Dickinson and Company, Sparks, MD). After 18 to 24 h’ incubation at 35 °C ± 2 °C in ambient air, plates were observed and suspicious colonies were identified by Matrix Assisted Laser Desorption Ionization –Time Of Flight (MALDI-TOF MS; Bruker Daltonics, Germany). Isolates identified as *Staphylococcus aureus* were tested with a 30 μg cefoxitin disk (Oxoid, Basingstoke, Hampshire, UK) on Mueller-Hinton agar (MHA) (BD Diagnostic Systems) and incubated for 18 to 24 h at 35 °C ± 2 °C in ambient air according to European Committee on Antimicrobial Susceptibility Testing (EUCAST) methodology [12]. Isolates with a zone diameter measuring < 22 mm were reported as MRSA as per EUCAST guidelines [13].

### Acute care hospital

The hand hygiene programme was instituted in our ACH from 1 April 2016. Our ACH is a 700 bedded facility which houses 18 wards assigned to various specialties, a combined Intensive Care Unit and High Dependency Unit of 72 beds, our main operating theatre complex, our renal dialysis unit as well as our outpatient clinics. We have no paediatric, obstetric and gynaecology, haematology, oncology or organ transplant services. There were 34,920 and 41,612 admissions in 2016 and 2017 respectively. We have a universal active surveillance testing (AST) programme for MRSA: all admissions not known to be previously positive for MRSA are swabbed, as well as all discharges. Transmission is assessed to have occurred if the discharge swab turns positive.

### Community care hospital

We instituted the same hand hygiene programme in our CCH on the 1 April 2017. Our CCH houses 7 wards that offer step down care in the form of dementia, rehabilitation and palliative care in addition to stepdown subacute care from our ACH. There were 2194 and 2863 admissions in 2016 and 2017 respectively. The MRSA surveillance is similar to that described in the ACH, however, due to increased length of stay of these patients, an additional 14th day MRSA swab is collected for all patients negative for MRSA on admission to the CCH.

Surveillance data for hospital acquired MRSA bacteraemia (using NHSN criteria) and MRSA transmission rates (based on those who were positive on exit swabs after negative entry swabs) were trended with our Hand Hygiene rates to help assess the impact of the programme for both the ACH and CCH.

Surveillance data for hospital-onset Clostridioides difficile (HO-CD), multi-resistant gram-negative bacilli including carbapenem resistant enterobactereciae (CRE) and also vancomycin resistant enterococci (VRE) (using NHSN criteria) was also recorded during that time frame.

### Statistical methods

Based on the data collected, Mann Whitney/Wilcoxon rank sum test was used to see if there was any significant differences in the hand hygiene compliance rates between the study time period.

## Results

Overall, we analysed the data from July 2015 to December 2017.

Acute care hospital.

There were 21,582 hand hygiene observations over 11 wards initially in 2015 which increased to 18 wards by the end of 2017. In 2015, our hand hygiene compliance rates had plateaued at 67% across the hospital. We observed a steady increase from 65 to 72% in 2016 since the commencement of the project in April 2016. This increase was sustained and carried on into 2017 where we saw a mean improvement of 6% from the previous year to 78%;. These increases were seen in both the scores of ICNs and “Secret Shoppers” with 6 and 10% mean improvement in hand hygiene compliance. The *t*-value pre-intervention vs post intervention analysed up to Dec 2017 was 5.25 with a statistically significant *p*-value < 0.00001.

**Fig. 2:**
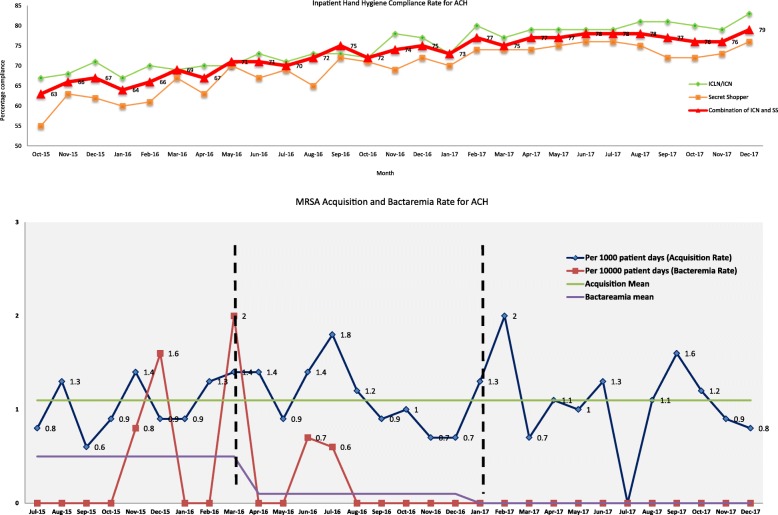
Hand Hygiene, MRSA bacteraemia and MRSA transmission rates for the ACH

**Fig. 3:**
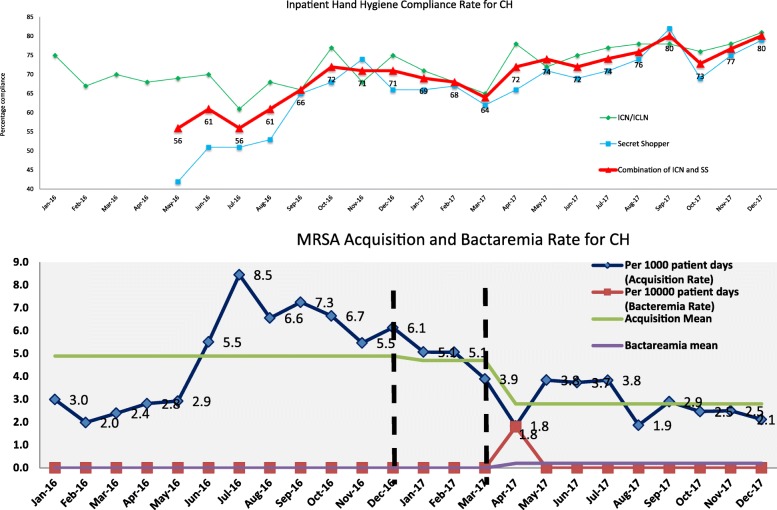
Hand Hygiene, MRSA bacteraemia and MRSA transmission rates for the ACH

Our MRSA bacteraemia rates also saw a significant reduction as detailed in Fig. 3. We reported no MRSA bacteraemia at our institution in 2017 from 5 in 2016. Again the *t*-value pre-intervention vs post intervention analysed up to Dec 2017 was 2.34 with a statistically significant *p*-value < 0.013. Our overall average MRSA transmission rate was at 1.1 per 1000 patient days before April 2016 and remained at 1.1 per 1000 patient days for the rest of 2016 and 2017. Our prevalence rate of MRSA was 5.8% in 2016 and 2017. Compliance rates of collection of AST swabs was 95% (Fig. 2).

HO-CD rates for 2016 was 3.3 per 10,000 patient days, with rates of 2.6 per 10,000 patient days from January to April (pre-intervention) and 3.6 per 10,000 patient days from May to December (post intervention). HO-CD rates for 2017 was 2.5 per 10,000 patient days. There were no cases of clustering or transmission identified. There was no statistically significant difference between 2016 and 2017, nor between the pre-intervention of post intervention period in 2016 in HO-CD rates. The numbers of CRE and VRE were too small to do meaningful statistical considerations.

From April 2016 to December 2017, out of 14 participating wards, 11 wards at some point in the year fell into the “two consecutive month below target” category, and 6 wards fell into the “three months below target” category. However, no task forces were actually established in these wards as rates improved by the time the task forces were convened. Targets were revised and increased by 10% of the mean from the previous year for 2017. Out of 18 participating wards, 14 wards fell into the “two months below target” category. Nine wards fell into the “three months below target” category.

### Community care hospital

There were 5770 hand hygiene observations over 5 wards initially in 2015, up to 7 wards by the end of 2017. The results were similar to those observed in the ACH. (Figure 3) we observed a steady increase in hand hygiene compliance from a mean of 64 to 75% since the initiation of the model when using the composite scores of ICNs and Secret Shoppers. The *t*-value pre-intervention vs post-intervention was 4.96 with statistically significant *p*-value of 0.00005. Again this trend was preserved when ICNs and “Secret Shopper” data were independently collected. There was only 1 bacteraemia reported in April 2017 at the community hospital. The MRSA transmission rate before May 2017 was 5.72 per 1000 patient days, after commencement of the project, a sustained decrease to 2.79 per 1000 patient days was observed. (Figure 3) the *t*-value pre-intervention vs post-intervention was 5.125 with statistically significant *p*-value of 0.00035. MRSA admission swab prevalence rates were 13.1% for 2016 and 20.6% in 2017. Our compliance rates to AST were 90%.

HO-CD rates for 2016 was 2.0 per 10,000 patient days. HO-CD rates for 2017 was 2.5 per 10,000 patient days with rates of 1.8 per 10,000 patient days from January to April (pre-intervention) and 2.9 per 10,000 patient days from May to December (post intervention). There were no cases of clustering or transmission identified. There was no statistically significant difference between 2016 and 2017, nor between the pre-intervention of post intervention period in 2017. The numbers of CRE and VRE were too small to do meaningful statistical considerations.

From April 2017 to December 2017, out of 7 wards, 6 wards fell into the “two month below target” category, and 4 wards fell into the “three months below target” category.

A summary of the results for the acute care hospital (ACH) and community hospital (CH) are presented in Table 1.

**Table 1:** Summary of results for ACH and CH

Acute care hospital	Pre-intervention Oct 2015-Mar 2016	Post intervention Apr 2016-Dec 2017	*p* value
Hand Hygiene Rate	65.89	73.78	< 0.00001
MRSA bacteremia	0.73	0.06	< 0.001
MRSA transmission rate per 1000 patient days	1.13	1.09	NS
Community Hospital	Pre-intervention May 2016-Mar 2017	Post-intervention Apr 2017-Dec 2017	*p* value
Hand Hygiene rate	64.26	74.95	0.00005
MRSA bacteremia	0	0.204	NS
MRSA transmission rate per 1000 patient days	5.72	2.79	0.00035

There was a cluster of CRE cases that was detected in June 2017 that resulted in stepped up environmental cleaning and increased hand hygiene audits in the CH, so some of the improvement cannot be ascribed to the introduction of the accountability model alone.

## Discussion

We saw sustained improvement in Hand Hygiene rates which we believe were the results of the intervention. As the numbers were small, it is hard to interpret MRSA bacteraemia rates and MRSA transmission rates.

Several papers have described personal accountability models describing punitive measures or reward systems that seem to have worked [8, 14]. Most have involved identification of staff to an individual level during Hand Hygiene audits. There are several problems with this approach including losing the ability to do “secret shopper” audits and questions of fairness in targeting.

Our approach is a hybrid of location specific accountability models and individual specific accountability models. We do note that the impact was seen most clearly on the nursing staff and less so from the doctors and other allied health staff. This is probably due to the fact that most nurses are permanently deployed to specific wards while senior doctors tend to rotate through several wards. The impact on transmission was not as acutely observed as the baseline numbers were relatively low [7–9, 14–16].

Interestingly, as part of the programme, if a ward had accumulated a third month of non-compliance with their hand hygiene target, according to our protocol, the ward would have been asked to form a task force to improve rates. In reality, we found that this was not necessary. Most wards were already concerned with not being able to achieve their targets by the second month and had already instituted projects for improvement on their own and in every case, this resulted in an improvement in hand hygiene compliance so no ward was below target four months in a row.

This project also highlights the importance of assigning realistic targets with modalities of negotiation and supportive autonomy to wards in the methods they wanted to deploy to improve their hand hygiene rates. Often when targets are set at a hospital wide level, some areas that are too far below these targets may view the exercise as futile and give up even before the initiative commences. The “sweet spot” for a target which is neither too high to be unattainable or too low to be mediocre is challenging and we adopted a 10% increase above the baseline which seems to have worked, but perhaps we could be more ambitious the next time around [17–19]. Adequately set goals create a lot of energy and momentum within an organization which is what we observed at our institution [17].

There are a number of limitations to this report. We were not able to document a decrease in MRSA transmission rates but this may be because of limitations of a single swab at entry and exit.

The time frame of this report includes a period of transition, where we moved from a small 300 bed facility to a new campus totalling 1100 beds. The improvements seen in our rates may have been due to staff gaining experience and familiarity with our new environment. However, this improvement was sustained over 2 years and continues to the time of writing of this report. The impact of environmental contamination as occurs in new facilities over time as evidenced by studies done in other institutions was also not taken into account [20]. In spite of this and MRSA prevalence rates remaining the same in the ACH and increasing in the CCH, we did not see an increase in MRSA transmission in the ACH and saw a decrease in MRSA transmission in the CCH which we think argues favourably for the intervention. A caveat to this however, is that there was a cluster of CRE identified on one ward in our CCH that may have resulted in improved hand hygiene rates but believe that the impact on the overall CCH hand hygiene rate was limited as it was was contained to a single ward.

## Conclusions

Using a ward level, as opposed to, individualised accountability for hand hygiene is possible and can be successful in improving hand hygiene rates, possibly reducing transmission of MDROs. Realistic targets need to be set and adequate rewards and incentives provided to ensure continuous improvement and ownership of the programme by the various wards.

## Data Availability

All data generated and analysed during this study are included in the published article.
